# Total flavonoids extracted from *Penthorum chinense* Pursh mitigates CCl_4_-induced hepatic fibrosis in rats via inactivation of TLR4-MyD88-mediated NF-κB pathways and regulation of liver metabolism

**DOI:** 10.3389/fphar.2023.1253013

**Published:** 2023-11-23

**Authors:** Sujuan Wang, Wenqing Li, Wenxiu Liu, Lei Yu, Fu Peng, Junyuan Qin, Lin Pu, Yunli Tang, Xiaofang Xie, Cheng Peng

**Affiliations:** ^1^ State Key Laboratory of Southwestern Chinese Medicine Resources, School of Pharmacy, Chengdu University of Traditional Chinese Medicine, Chengdu, China; ^2^ Key Laboratory of Drug-Targeting and Drug Delivery System of the Education Ministry and Sichuan Province, Sichuan Engineering Laboratory for Plant-Sourced Drug and Sichuan Research Center for Drug Precision Industrial Technology, West China School of Pharmacy, Sichuan University, Chengdu, China

**Keywords:** *Penthorum chinense* Pursh, total flavonoids, hepatic fibrosis, inflammation, liver metabolomics, TLR4/MyD88/NF-κB pathways

## Abstract

**Background:** *Penthorum chinense* Pursh (PCP) is widely utilized in China to treat a variety of liver diseases. It has been shown that flavonoids inhibit inflammation and have the potential to attenuate tissue damage and fibrosis. However, the mechanisms underlying how total flavonoids isolated from PCP (TFPCP) exert their anti-fibrotic effects remain unclear.

**Methods:** The chemical composition of TFPCP was determined using UHPLC–Q-Orbitrap HRMS. Subsequently, rats were randomly assigned to a control group (Control), a carbon tetrachloride (CCl_4_)-induced hepatic fibrosis model group (Model), a positive control group [0.2 mg/(kg∙day)] of Colchicine), and three TFPCP treatment groups [50, 100, and 150 mg/(kg∙day)]. All substances were administered by gavage and treatments lasted for 9 weeks. Simultaneously, rats were intraperitoneally injected with 10%–20% CCl_4_ for 9 weeks to induce liver fibrosis. At the end of the experiment, the liver ultrasound, liver histomorphological, biochemical indicators, and inflammatory cytokine levels were tested respectively. The underlying mechanisms were assessed using Western blot, immunohistochemistry, immunofluorescence, RT-qPCR, and metabolomics.

**Results:** Fourteen flavonoids were identified in TFPCP. Compared with control animals, CCl_4_-treated rats demonstrated obvious liver injury and fibrosis, manifested as increases in gray values, distal diameter of portal vein (DDPV) and a decrease in blood flow velocity (VPV) in the ultrasound analysis; increased biochemical index values (serum levels of ALT, AST, TBIL, and ALP); marked increases in the contents of fibrotic markers (PC III, COL4, LN, HA) and inflammatory factors (serum TNF-α, IL-6, and IL-1β); and significant pathological changes. However, compared with the Model group, the ultrasound parameters were significantly improved and the serum levels of inflammatory cytokines were reduced in the TFPCP group. In contrast, the expression of TGF-β_1_, TLR4, and MyD88, as well as the p-P65/P65 and p-IκBα/IκBα ratios, were considerably reduced following TFPCP treatment. In addition, we identified 32 metabolites exhibiting differential abundance in the Model group. Interestingly, TFPCP treatment resulted in the restoration of the levels of 20 of these metabolites.

**Conclusion:** Our findings indicated that TFPCP can ameliorate hepatic fibrosis by improving liver function and morphology via the inactivation of the TLR4/MyD88-mediated NF-κB pathway and the regulation of liver metabolism.

## 1 Introduction

Hepatic fibrosis, the primary pathological characteristic of prolonged liver disease, results from the continuous repair of liver injury and persistent inflammation. During hepatic fibrogenesis, there is a gradual buildup of fibrillar extracellular matrix (ECM) and the formation of nodules in the liver parenchyma (Bottcher and Pinzani, 2017). Without intervention, extensive fibrosis eventually progresses to cirrhosis and even liver cancer, both of which can be fatal (Berumen et al., 2021). Cirrhosis is associated with high morbidity and mortality and ranks as the 11th leading cause of death globally (Asrani et al., 2019; Wang et al., 2021). Nevertheless, liver fibrosis can be reversed before it progresses to cirrhosis (Yu et al., 2019), highlighting the importance of prevention and early treatment for this condition.

Tissue damage and inflammation are two key triggers for fibrosis and regeneration in the liver. Kupffer cells and hepatic stellate cells (HSCs) are the major sources of ECM in liver fibrosis, and both are essential components in hepatic fibrogenesis and targets of pro-inflammatory mediators (Seki et al., 2007). Carbon tetrachloride (CCl_4_) is extensively used to generate animal models of liver fibrosis owing to the reproducibility and efficiency of its effects. Moreover, the pathological alterations observed in these models closely resemble those seen in chronic hepatitis and hepatic fibrosis in humans (Liedtke et al., 2013). First, the metabolism of CCl_4_ mediated by the cytochrome P450-dependent mixed-function oxidase system generates active trichloromethyl radicals (CCl_3_•) and chlorine radicals (Cl•) (Zhang et al., 2018). This leads to lipid peroxidation and the subsequent solubilization of cell membranes, which can result in liver cell injury or death and, consequently, liver tissue damage. Inflammation results in cell death and *vice versa*, a process that has been termed “necroinflammation” (Mack, 2018). Specifically speaking, CCl_4_ direct damage to hepatocytes, when hepatic cells are injured, neighboring liver cells, including Kupffer cells, produce pro-inflammatory cytokines, such as tumor necrosis factor-alpha (TNF-α) and interleukin 6 (IL-6), which can activate HSCs in a paracrine manner; activated HSCs are subsequently stimulated by both autocrine and paracrine signals pathways thereby driving the development of fibrosis (Liu et al., 2021; Wang et al., 2021). Meanwhile, inflammation in the liver acts as a further trigger for the activation of HSCs and their differentiation from quiescent cells into myofibroblasts, whereby they acquire proliferative, pro-inflammatory, and contractile properties (Sato et al., 2014). Colchicine protects from CCl_4_-induced liver damage based mostly on the stimulation of repair by its mitogenic activity (Weber et al., 2003). Secondly, the liver is a primary target of intestine-derived bacterial products, and the incidence of bacterial translocation has been shown to increase in several models of liver disease, and leading to an increase in the levels of profibrogenic Toll-like receptor 4 (TLR4) agonists, such as LPS, in hepatic fibrosis (Seki et al., 2007). Both *in vitro* and *in vivo* studies have shown that TGF-β is a key modulator of HSC activation. Kupffer cells can produce large amounts of TGF-β, thereby promoting HSC activation and fibrogenesis, while TLR4 facilitates HSC activation by exposing the cells to Kupffer cell-derived TGF-β, which renders them more sensitive to this cytokine (Seki et al., 2007). These observations suggest that the suppression of liver inflammation may slow or even prevent the progression of hepatic fibrosis.

The medicinal plant *Penthorum chinense* Pursh (PCP; family: Saxifragaceae) is a well-known traditional Chinese medicine (TCM) that has been used for the treatment of liver disease since the Ming era (1400s) (Zeng et al., 2013; Wang et al., 2020). In modern times, PCP has been commercially developed (“Gansu” granules, capsules, and pills) for the treatment of chronic active hepatitis, hepatitis B, and different forms of acute viral hepatitis (Yin et al., 2020). Flavonoids are a group of substances with a C6-C3-C6 backbone structure found widely in the plant kingdom (Serafini et al., 2010). Studies have shown that these chemicals can block regulatory enzymes or transcription factors that are crucial for regulating inflammatory mediators, and also have the potential to attenuate tissue damage or fibrosis via their potent anti-oxidative properties and their ability to inhibit stellate cell activation (Serafini et al., 2010; Wang et al., 2020). Flavonoids are among the primary bioactive components of PCP; however, it is still unknown whether total flavonoids isolated from PCP (TFPCP) play a substantial role in the anti-inflammatory and anti-fibrotic effects of PCP.

Because of the complexity of their components and their synergistic actions, it has proven extremely challenging to elucidate the mechanisms of action of TCMs (Beyoglu and Idle, 2020). Metabolomics represents a key approach for overcoming this difficulty and has shown great promise in bridging the gap between TCM and molecular pharmacology (Wang et al., 2005). Metabolomics is an emerging technique in systems biology for the identification of changes in holistic metabolic profiles in biological systems. Additionally, this technique represents a comprehensive quantitative and qualitative approach to investigating how metabolites interact with key environmental factors *in vivo* (Shu et al., 2020). Metabolomics has been utilized to describe the diverse physiological and pathological states of organisms in response to exogenous physical, chemical, and environmental stimuli (Chang et al., 2017). Over recent years, metabolomics has been widely employed in the evaluation and tracking of disease processes and has offered significant insights into the pathophysiology of a variety of conditions (Zhao et al., 2016). Importantly, several studies have reported shifts in host metabolism during the development of liver fibrosis, and have suggested that regulating the metabolism of the host may be one strategy for alleviating hepatic fibrosis (Zhang et al., 2018; Yang et al., 2021). For instance, compared with healthy controls, patients with hepatic fibrosis displayed significantly altered carbohydrate, lipid, and amino acid serum metabolism (Yoo et al., 2019). Given these observations, we postulated that TFPCP therapy may alter the metabolic profile and thus alleviate the systemic state of hepatic fibrosis. Accordingly, we performed a metabolomic analysis of liver tissue to identify which endogenous metabolites and biological processes are affected by TFPCP to regulate liver metabolism.

The objective of this study was to evaluate the therapeutic effect of TFPCP on hepatic fibrosis *in vivo* using a CCl_4_-induced rat model of the disease, as well as clarify the potential underlying molecular processes employing pharmacodynamic and metabolomic approaches.

## 2 Materials and methods

### 2.1 Reagents and chemicals

CCl_4_ (RH298281) was purchased from Luoen Chemical Reagent Co., Ltd. (Shanghai, China). Olive oil (J2122345) was purchased from Shanghai Aladdin Biochemical Technology Co., Ltd. (Shanghai, China). Colchicine (20200908) was bought from Yunnan Phytopharmaceutical Co., Ltd. (Yunnan, China). D101 macroporous adsorption resin (2020101901) was purchased from Chengdu Kelong Chemical Reagent Factory (Chengdu, China). The reagents for measuring aspartate aminotransferase (AST, 552616), alanine aminotransferase (ALT, 561236), alkaline phosphatase (ALP, 568781), total bilirubin (TBIL, 548537), serum albumin (ALB, 551772), and total protein (TP, 553813) levels were obtained from Roche (Basel, Switzerland). Rat procollagen III (PC III, R544UBAE9R), collagen type Ⅳ (COL4, RDTFJW7MYX), hyaluronidase (HA, 6E6UQPEADI), TNF-α (CHHNV14X86), IL-6 (2ULD7M9LKF), IL-1β (MKIMB94QHN), and transforming growth factor-beta 1 (TGF-β_1_, AK03420T5560) ELISA Kits were purchased from Wuhan Elabscience Biotech Co., Ltd. (Wuhan, China). The anti-laminin antibody (LN, 031807CYF137370321) was obtained from Shanghai JONLN Reagent Co., Ltd. (Shanghai, China). Anti-TLR4 (AC220407057) and anti-P65 (AC220429001) antibodies were obtained from Wuhan Servicebio Technology Co., Ltd. (Wuhan, China). The anti-myeloid differentiation factor 88 antibody (MyD88, GR3356289-17) was obtained from Abcam, Inc. (Cambridge, United Kingdom). Anti-p-P65 (19u7497), anti-IκBα (80k0141), and anti-p-IκBα (47y8501) antibodies were purchased from Qingke Biotech Co., Ltd. (Jiangsu, China).

### 2.2 Preparation of the TFPCP extract and chemical component identification

PCP herbal slice was provided by Sichuan Gulin Gansu Pharmaceutical Co., Ltd. and was identified by Assoiate Professor Jihai Gao of Chengdu University of Traditional Chinese Medicine as *Penthorum chinense* Pursh of the genus *Penthorum Gronov. ex L* in the family Saxifragaceae. TFPCP were obtained as follows: Briefly, desiccated, above-ground portions of PCP were heated with distilled water under reflux for 2 h. After filtering, the residue was extracted a second time under identical conditions. The extracts were combined and condensed under reduced pressure to a concentration of 4 g/mL, applied to D101 macroporous resin, and eluted with 60% aqueous ethanol (aq. EtOH). The 60% aq. EtOH eluate was collected and concentrated *in vacuo* to yield a residue, with the final residue yield being the TFPCP. The percentage content of total flavonoids in PCP was determined by UV spectrophotometry at 510 nm, with rutin serving as the reference. The chemical components of TFPCP were identified using ultra-high performance liquid chromatography coupled with hybrid quadrupole-orbitrap high-resolution mass spectrometry (UHPLC–Q-Orbitrap HRMS). Separation was performed on an Accucore C18 column (3 mm × 100 mm, 2.6 μm; Thermo Scientific, Rockford, United States). The mobile phase was 0.1% formic acid (A) and 0.1% formic acid in acetonitrile (B). The elution gradient was 0–10 min, 6%–18% B; 10–20 min, 18%–35% B; 20–30 min, 35%–55% B; 30–40 min, 55%–85% B; 40–45 min, 85%–95% B; and 45–50 min, 95%–99% B; the flow rate was 0.3 mL/min.

### 2.3 Animals and treatments

**FIGURE 1:**
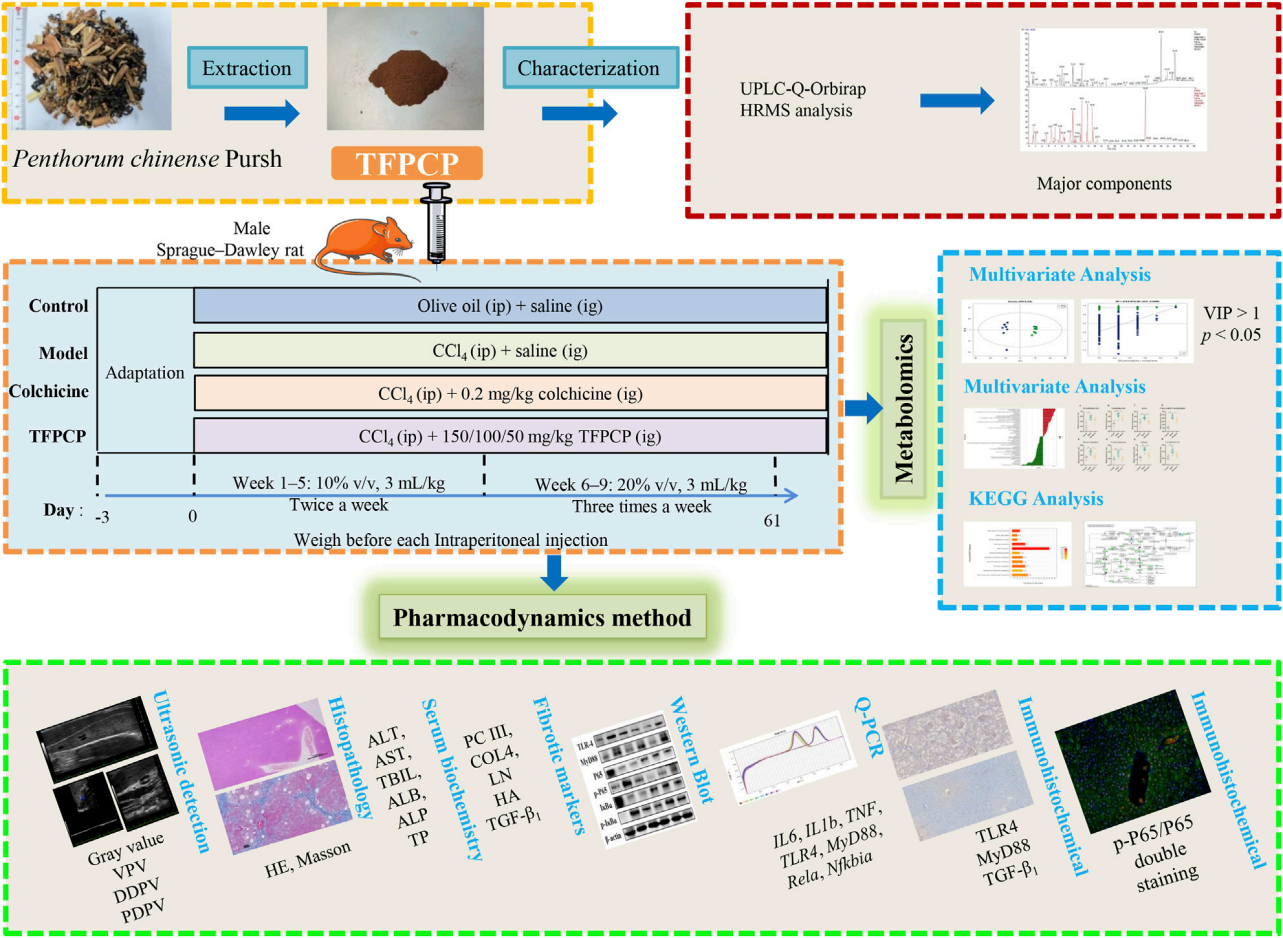
The schematic diagram of the animal and total experimental design.

Forty-eight male Sprague–Dawley rats, weighing 180–220 g, were procured from SPF (Beijing) Biotechnology Co., Ltd. (NO. SCXK 2019-0010) and maintained under controlled conditions (temperature: 20°C–25°C; relative humidity: 60% ± 5%; 12-h light/12-h dark photoperiod) and with free access to food and water. All animal experiments were approved by the Animal Ethics Committee of Chengdu University of Traditional Chinese Medicine (No. 2021-69). Following 3 days of acclimation, the rats were randomly divided into the following six groups (*n* = 8/group): A control group (Control); a CCl_4_-induced hepatic fibrosis model group (Model); a CCl_4_ + colchicine group (Colchicine, 0.2 mg/(kg∙day) (Kershenobich et al., 1988; Zhang et al., 2016); and three CCl_4_ + TFPCP groups [50, 100, and 150 mg/(kg∙day), respectively]. Animals in both the Control and Model groups were administered saline intragastrically (10 mL/kg) for 9 weeks, while those in the Colchicine and CCl_4_ + TFPCP groups were treated intragastrically with the same volume of saline containing the respective treatments continuously for 9 weeks. Meanwhile, olive oil was administered intraperitoneally to rats in the Control group (3 mL/kg), while rats in the other five groups received an intraperitoneal injection of olive oil containing CCl_4_ (3 mL/kg) of differing concentrations (week 1 to week 5: 10% v/v, 3 mL/kg; week 6 to week 9: 20% v/v, 3 mL/kg). Olive oil or CCl_4_ (to induce hepatic fibrosis) was given twice a week in the first 5 weeks and three times a week in the following 4 weeks. After 9 weeks of treatment, the rats were anesthetized with 40 mg/kg sodium pentobarbital, and serum and liver tissue were collected for further experiments. A schematic diagram of the animal and total experimental design is shown in Figure 1.

### 2.4 Liver ultrasound

On the last 3 days, to evaluate the status of the liver parenchyma and the lumen of the hepatic veins, rats were sedated through the inhalation of 4% isoflurane (RWD Life Science Co., Ltd., China) and subjected to ultrasonography. Liver ultrasound was conducted on a Vevo 3100 Preclinical Imaging System (FujiFilm VisualSonics, Canada) using a MX-250 probe (14–28 MHz). The gray value, the proximal diameter of portal vein (PDPV), the distal diameter of portal vein (DDPV), and the blood flow velocity (vein peak velocity [VPV]) were determined as an assessment of liver function.

### 2.5 Hepatic histopathological examination

After macroscopic examination, liver tissues were fixed in 4% paraformaldehyde, desiccated, embedded in paraffin wax, cut into 4-µm-thick sections, and subjected to hematoxylin and eosin (H&E) and Masson staining according to standard procedures. Images were captured using a digital slide scanner (NanoZoomer-S60, Hamamatsu, Japan). Three random fields were taken and scoring by a blinded experimenter. The relative collagenous fiber areas were detected by Masson staining and quantified with the Image-Pro Plus 6.0 software. The fibrotic area (%) was calculated according to the following formula: DAB staining area/total image area × 100%.

### 2.6 Determination of serum biochemical indexes

To assess liver function, serum levels of ALT, AST, TBIL, ALB, ALP, and TP were measured using an automatic biochemical analyzer (Roche).

### 2.7 Measurement of serum inflammatory cytokine and fibrotic marker levels

At the end of 9 weeks, the serum levels of the inflammatory cytokines TGF-β_1_, TNF-α, IL-6, and IL-1β and the fibrotic markers PC III, COL4, LN, and HA were assessed using the respective ELISA kits following the manufacturer’s instructions.

### 2.8 Metabolomics analysis

Metabolomic analysis was carried out using the Agilent 1290 Infinity LC System (Agilent, United States) and the AB Triple TOF 6600 system (AB SCIEX, United States). Separation was performed on an ACQUITY UPLC BEH C18 column (2.1 mm × 100 mm, 1.7 µm) (Waters, Ireland). The mobile phase was 25 mM ammonium acetate and 25 mM ammonium hydroxide [v/v] in water (A) and acetonitrile (B) and the separation program was as follows: 0–1 min, 15:85, v/v; 1–11 min, gradient increase from 15:85 to 35:65, v/v; 11–11.1 min, gradient increase from 35:65 to 60:40, v/v; 11.1–15.1 min, 60:40, v/v; 15.1–15.2 min, gradient decrease from 60:40 to 15:85, v/v; 15.2–20.2 min, 15:85, v/v. The flow rate was 0.4 mL/min and *the* autosampler temperature was 5°C. Sample analysis was performed in both negative and positive ionization modes. For electrospray ionization (ESI), the source temperature was 600°C and the IonSpray Voltage Floating (ISVF) was ±5500 V.

### 2.9 Western blot

For protein extraction, liver tissue was homogenized in RIPA lysis buffer. Protein concentrations were determined using BCA Protein Assay Kits (Thermo Scientific, Rockford, United States). Protein samples were mixed with SDS sample buffer and heated to 100°C for 10 min. Subsequently, equal amounts of protein were separated by 8%–10% SDS–polyacrylamide gel electrophoresis, electro-blotted onto PVDF membranes, blocked with bovine serum albumin (BSA) for 2 h, and then incubated first with primary antibodies against TLR4, MyD88, P65, IκBα (1:1,000), p-P65, and p-IκBα (1:800) overnight at 4°C and, after washing, with the respective secondary antibodies (1:10,000). The immunoreactive bands were developed using chemiluminescence and the gray values were evaluated using ImageJ software.

### 2.10 RNA isolation and RT-qPCR analysis

Total RNA was extracted from liver tissue using Trizol reagent (Beyotime, Shanghai, China) according to the manufacturer’s instructions. The extracted RNA was dissolved in RNase-free water and the RNA concentration was measured by spectrophotometry. Total RNA (1 μg) was reverse transcribed into cDNA (Thermo Scientific, Rockford, United States), following which qPCR was performed using standard TB Green Premix Ex Taq (Takara, Osaka, Japan) on a real-time PCR detection system from Rocgene (Beijing, China). β-actin served as an internal reference. The sequences of the primers used are shown in Table 1 (Designed and synthesized by Beijing Tsingke Biotech Co., Ltd.). The primers are all designed on exons.

**TABLE 1:** Primer sequences used in RT-qPCR analyses.

Primer	Primer sequence	Gene ID	Amplified product size
*TNF*	F:CATCCGTTCTCTACCCAGCC	24835	146bp
	R:AATTCTGAGCCCGGAGTTGG		
*IL6*	F:TCCTACCCCAACTTCCAATGC	24498	73bp
	R:GGTCTTGGTCCTTAGCCACT		
*IL1b*	F:GACTTCACCATGGAACCCGT	24494	200bp
	R:CAGGGAGGGAAACACACGTT		
*TLR4*	F:TCCAGAGCCGTTGGTGTATC	29260	198bp
	R:AGAAGATGTGCCTCCCCAGA		
*MyD88*	F:CTCGCAGTTTGTTGGATGCC	301059	119bp
	R:CTCGATGCGGTCCTTCAGTT		
*Rela*	F:TGTATTTCACGGGACCTGGC	309165	110bp
	R:CAGGCTAGGGTCAGCGTATG		
*Nfkbia*	F:CTCAAGAAGGAGCGGTTGGT	25493	184bp
	R:CCAAGTGCAGGAACGAGTCT		
*ACTB*	F:AGATCAAGATCATTGCTCCTCCT	81822	174bp
	R:ACGCAGCTCAGTAACAGTCC		

### 2.11 Immunohistochemistry

Paraffin-embedded sections were dewaxed, rehydrated, subjected to antigen retrieval, and incubated with primary antibodies targeting TLR4 (1:1,000) and MyD88 (1:1,200) at 4°C overnight. After washing, the samples were incubated with secondary antibody (1:200) at ambient temperature for 1 h, washed, stained with DAB, stained with hematoxylin, dehydrated through a graded ethanol series, cleared with xylene, and mounted with neutral rubber. Images were captured using a digital slide scanner (NanoZoomer-S60). Three random fields were taken and scoring by a blinded experimenter. The TGF-β_1_, TLR4 and MyD88 relative IOD in liver were analyzed using ImageJ software. The relative IOD was calculated according to the following formula: lg (255/mean gray value).

### 2.12 Immunofluorescence staining

Sections were deparaffinized, rehydrated, subjected to antigen retrieval, blocked in blocking buffer containing 3% BSA for 30 min, and incubated with primary antibodies against P65 (1:200) and p-P65 (1:500) overnight at 4°C. After washing, the sections were incubated with secondary antibody for 1 h at ambient temperature, counterstained with DAPI. Photographs were blindly taken at three random fields under a fluorescence microscope (Nikon Eclipse C1, Tokyo, Japan), and scoring by a blinded experimenter.

### 2.13 Statistical analysis

Principal component analysis (PCA) and orthogonal partial least squares discriminant analysis (OPLS-DA) were used to analyze metabolic alterations. Variable importance in projection (VIP) values > 1 and *p*-values <0.05 were considered significant. In addition, association and pathway analyses of the differentially abundant metabolites were conducted using online databases such as MetaboAnalyst, HMDB, and KEGG.

The data were analyzed in GraphPad Prism 8.0 and the results are presented as means ± SD. One-way ANOVA with Tukey’s *post hoc* test was used for comparisons among multiple groups. A *p*-value <0.05 was considered significant.

## 3 Results

### 3.1 Chemical analysis of TFPCP extracts

The percentage content of total flavonoids in the residue was found to be 53.8%. TFPCP extracts were analyzed using UHPLC–Q-Orbitrap HRMS. The acquired molecular masses and formulae were matched to the information contained in online databases (PubChem, MassBank Europe, MassBank of North America [MoNA], mzCloud Best Match, and mzVault Best Match) as well as to references. Then, a manual search and match was performed to determine which compounds belonged to which structural categories based on the precise MS/MS data. Finally, 14 flavonoids with various intensities were identified in TFPCP (Table 2; Supplementary Figure S1).

**TABLE 2:** Identification analysis of chemical component of TFPCP in ion mode of mass spectrometry.

No.	Identified compounds	Retention time (min)	Ionization Mode	Molecular formula	Detected (m/z)	Theoretical mass (m/z)	Error (ppm)	MS^2^ data (m/z)	References/Database ID
1	Quercetin-3β-D-glucoside	8.382	M-H	C21 H20 O12	463.08929	463.0882	−2.35376	301.03458,178.99768, 151.00310, 107.01246	Yang et al. (2021)
2	(+/−)-Taxifolin	8.638	M-H	C15 H12 O7	303.05157	303.05103	−1.78188	125.02362, 285.04053, 177.01894	MSBNK-RIKEN-PR309311 ^a^
3	Quercetin-3-O-pentoside	9.224	M-H	C20 H18 O11	433.07822	433.07763	−1.36234	433.07843	Amroun et al. (2021)
4	Quercetin	9.433	M-H	C15 H10 O7	301.03558	301.03538	−0.66437	178.99815, 151.00307, 107.01302	Yang et al. (2021)
5	Luteoloside	9.437	M-H	C21 H20 O11	447.09354	447.09329	−0.55917	151.00313, 107.01304	Yang et al. (2021)
6	Prunin	9.768	M-H	C21 H22 O10	433.11481	433.11402	−1.824	271.06146, 151.00299	Luo et al. (2021)
7	Kaempferol-3-O-arabinoside	10.26	M-H	C20 H18 O10	417.08353	417.08272	−1.94206	284.03284	Rescic et al. (2016)
8	Apigenin 7-O-glucoside	10.549	M-H	C21 H20 O10	431.09866	431.09837	−0.6727	151.00255, 107.01316	Yang et al. (2021)
9	Phloridzin	10.575	M-H	C21 H24 O10	435.13019	435.12967	−1.19505	273.07700, 123.04428, 119.04953	Chen et al. (2023)
10	Trilobatin	11.374	M-H	C21 H24 O10	435.13049	435.12967	−1.8845	273.07712	Xiao et al. (2017)
11	Pinocembrin	12.526	M + H	C15 H12 O4	257.08136	257.08084	−2.02271	153.01865	Simirgiotis et al. (2015)
12	Naringenin	14.192	M-H	C15 H12 O5	271.06171	271.0612	−1.88149	177.01903, 151.00305, 119.04947, 107.01301	Xiao et al. (2022)
13	Luteolin	14.56	M-H	C15 H10 O6	285.04086	285.04046	−1.40331	151.00316, 107.01313	Yang et al. (2021)
14	Cardamomin	24.522	M + H	C16 H14 O4	271.09723	271.09649	−2.72966	167.03436, 124.01624, 152.01097, 170.02182, 103.05477	CCMSLIB00000848351 ^b^

^a^ MassBank Europe.

^b^ MassBank of North America (MoNA).

### 3.2 TFPCP improved ultrasound parameters in the livers of rats with CCl_4_-induced hepatic fibrosis

**FIGURE 2:**
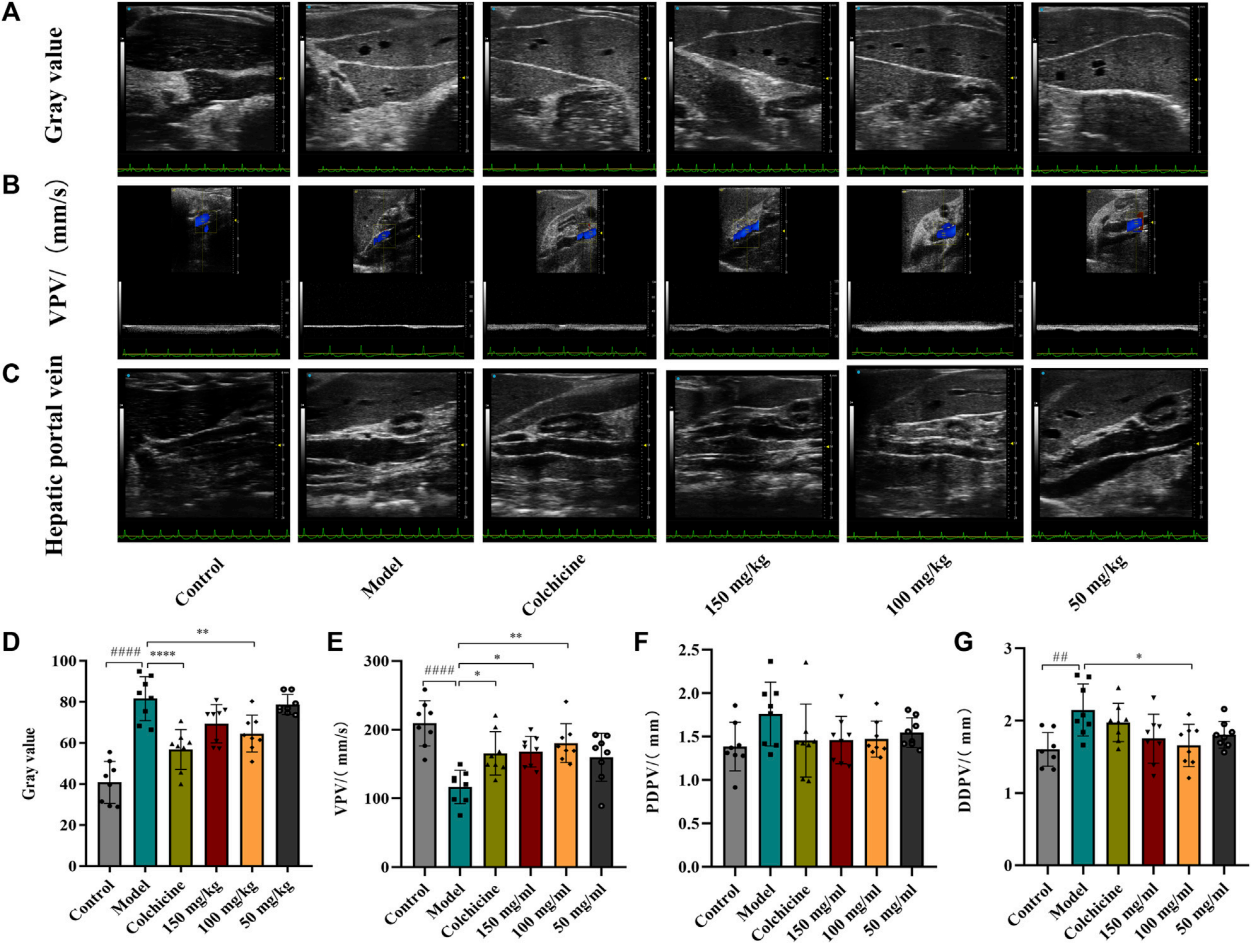
TFPCP improves ultrasonic Parameters of CCl_4_-induced hepatic fibrosis in rats. The ultrasonic detection of **(A, D)** Gray value, **(B, E)** blood flow velocity (VPV), **(C, F, G)** proximal diameter of portal vein (PDPV) and distal diameter of portal vein (DDPV). Values are presented as the means ± SD (*n* = 6). ^##^ *p* < 0.01 and ^####^ *p* < 0.0001 vs. control group; ^*^ *p* < 0.05, ^**^ *p* < 0.01 and ^****^ *p* < 0.0001 vs. model group.

Ultrasound was employed to examine the therapeutic effects of TFPCP on CCl_4_-induced hepatic fibrosis. Due to the pathology of hepatic fibrosis, there is a large amount of fibrous connective tissue hyperplasia and abnormal deposition of hepatic extracellular matrix in the confluent area and hepatic lobules leads to increased echogenicity of liver parenchyma. And the fenestrated structure of liver sinusoidal endothelial cell was damaged, which leads to the increase of hepatic sinusoids’ resistance to blood flow and the alteration of portal venous flow. This pathologic change in the hepatic sinusoids reduces the pressure gradient difference that maintains the normal blood supply to the portal vein, resulting in a slowing of portal venous blood flow velocity (vein peak velocity [VPV]) (Afdhal and Nunes, 2004). Meanwhile, in order to maintain the blood flow of the portal vein, the body causes the hepatic portal vein obstructive congestion to persist. Due to the greater compliance of the hepatic portal vein, it can adapt to large blood flow changes while the hepatic portal vein pressure changes little, which can lead to the widening of the internal diameter of the portal vein, such as PDPV and DDPV (Albrecht et al., 1999; Afdhal and Nunes, 2004). As depicted in Figure 2, compared with the Control group, the gray scale values and the DPVD were markedly increased in the Model group, whereas the VPV values were significantly decreased (*p* < 0.01). After the administration of TFPCP or colchicine, the gray values and DPVD were significantly decreased, while the VPV values were significantly increased; no differences in PPVD values were observed among the groups. These results indicated that both TFPCP and colchicine improved CCl_4_-induced liver fibrosis to some extent.

### 3.3 TFPCP ameliorated liver function and alleviated liver injury

**FIGURE 3:**
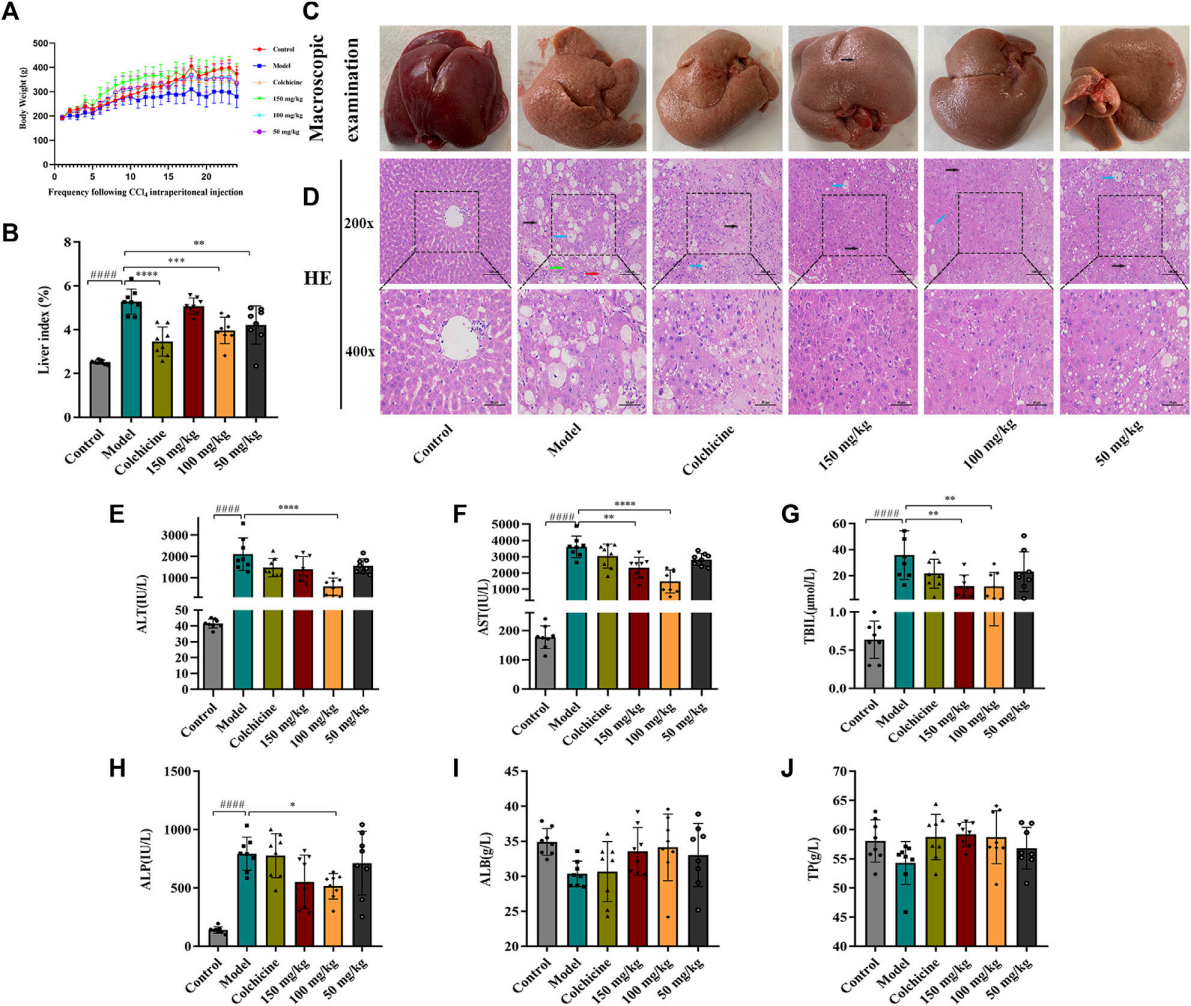
TFPCP can ameliorate liver function and alleviate liver injury. **(A)** Body weight (*n* = 8). **(B)** Liver index (*n* = 8). **(C)** Macroscopic examination of liver. **(D)** H&E staining (200×) and (400×) (*n* = 8). Black arrows indicate hepatocytes are steatosis, and circular vacuoles in cytoplasm. Blue arrows indicate hepatocyte swelling, necrosis, nuclear contraction, cytoplasmic vacuolation. Red arrows indicated infiltration of inflammatory cells. Green arrows indicate the thin fibrous septa that surrounded the hepatocytes to form pseudobullets of different shapes. **(E–J)** The ALT, AST, TBIL, ALP, ALB and TP levels in serum (*n* = 8). Values are presented as the means ± SD. ^####^ *p* < 0.0001 vs. control group; ^*^ *p* < 0.05, ^**^ *p* < 0.01 and ^****^ *p* < 0.0001 vs. model group.

Following 9 weeks of TFPCP administration, although the differences among the groups were not significant (Figure 3A), the body weight of rats in the Control, Colchicine, and CCl_4_ + TFPCP groups increased with time. Meanwhile, compared with the Control group, the biochemical index values (Figure 3B) were markedly increased in the liver tissues of rats in the hepatic fibrosis Model group; however, these changes were greatly attenuated with TFPCP treatment. Regarding anatomy, the liver tissue presented evident granules on the surface, was light in color and coarse in texture, and exhibited large-scale, small-particle formation and tissue swelling in the model groups (Figure 3C). H&E staining results showed that the livers of rats in the control group possessed a normal lobular architecture, complete with distinct central veins and radiating hepatic cords. In contrast, the liver tissue of animals in the Model group displayed disordered hepatic lobules, irregular hepatocyte arrangement, extensive hepatocyte swelling, necrosis, cytoplasmic vacuolization, thin fibrous septa that surrounded the hepatocytes to form pseudobullets of different shapes, and massive hepatocyte steatosis, round vacuoles of different sizes in the cytoplasm. However, TFPCP alleviated liver damage and improved the pathology of the liver to varying degrees (Figure 3D).

The contents of ALT, AST, TBIL, ALP, ALB, and TP in serum are frequently utilized as indicators of liver function. As shown in Figures 3E–J, the levels of ALT, AST, TBIL, and ALP in rats were greatly elevated in the presence of CCl_4_; however, the levels of these biochemical indices decreased considerably with TFPCP administration; no differences in TP and ALB concentrations were detected among the groups. Combined, these results demonstrated that although TFPCP treatment did not entirely prevent the development of liver injury, liver tissue morphology and transaminase levels were restored to varying degrees, indicating that TFPCP can alleviate liver damage.

### 3.4 TFPCP improved collagen deposition in the livers of rats with CCl_4_-induced hepatic fibrosis

**FIGURE 4:**
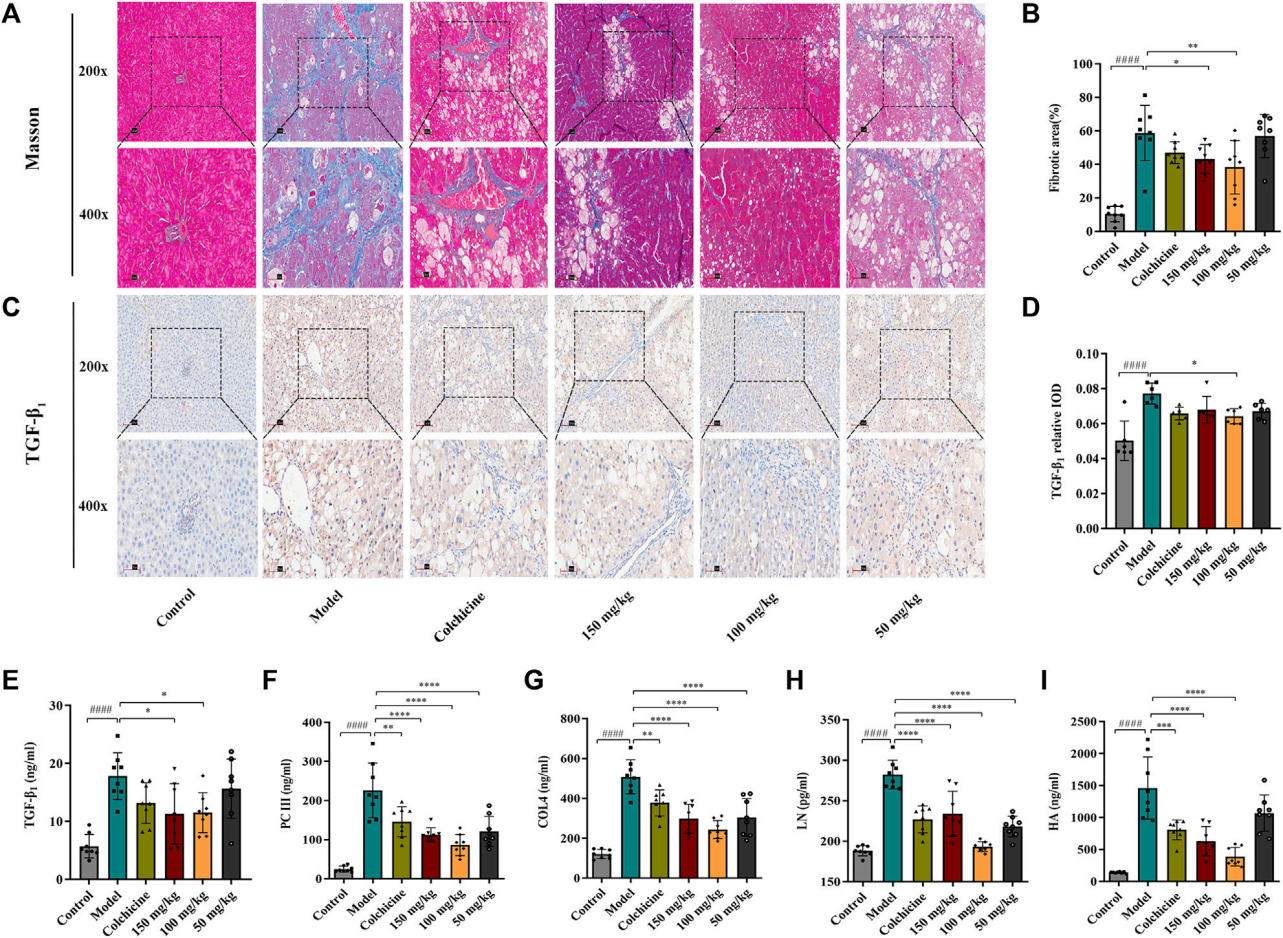
TFPCP improves the deposition of collagen. **(A, B)** Hepatic fibrosis assessed by masson staining (200×) and (400×) (*n* = 8). **(C, D)** TGF-β_1_ assessed by immunohistochemical (200×) and (400×) (*n* = 6). **(E)** Analysis of the serum TGF-β_1_ level (*n* = 8). **(F–I)** PC III, COL4, LN and HA fibrotic markers detection in serum (*n* = 8). Values were presented as the means ± SD. ^####^ *p* < 0.0001 vs. control group; ^*^ *p* < 0.05, ^**^ *p* < 0.01, ^***^ *p* < 0.001 and ^****^ *p* < 0.0001 vs. model group.

The results of the Masson staining revealed that collagen deposition was minimal in the Control group; in comparison, in the hepatic fibrosis Model group, there was a greater number of collagen fibers, dense collagen staining, and fibrotic scarring surrounding the central vein. Nevertheless, TFPCP treatment greatly attenuated collagen deposition and hepatic fibrosis induced by CCl_4_; moreover, the effect of TFPCP was better than that of colchicine (Figures 4A, B). TGF-β is a key mediator of HSC activation both *in vitro* and *in vivo* (Seki et al., 2007), while the activation of quiescent HSCs into myofibroblast-like cells is a crucial stage in hepatic fibrogenesis. Consequently, to further determine whether the inhibitory impact of TFPCP on collagen deposition was connected to HSC activation, we compared the expression of TGF-β_1_ among the groups. Immunohistochemical staining and serum ELISAs (Figures 4C–E) revealed that the level of TGF-β_1_ was significantly higher in rats of the Model group than in those of the Control group. However, compared with that seen in the model condition, TFPCP administration reduced the TGF-β_1_ staining intensity and serum TGF-β_1_ levels. Meanwhile, serum PC III, COL4, LN, and HA concentrations are important markers in hepatic fibrosis diagnosis. As shown in Figures 4F–I, CCl_4_ treatment substantially enhanced the serum concentrations of PC III, COL4, LN, and HA; however, the opposite effect was observed with TFPCP administration. These findings indicated that TFPCP can reduce collagen formation as well as prevent HSC activation.

### 3.5 The TLR4/MyD88/NF-κB signaling pathway was involved in the CCl_4_-induced inflammatory response in hepatic fibrosis

**FIGURE 5:**
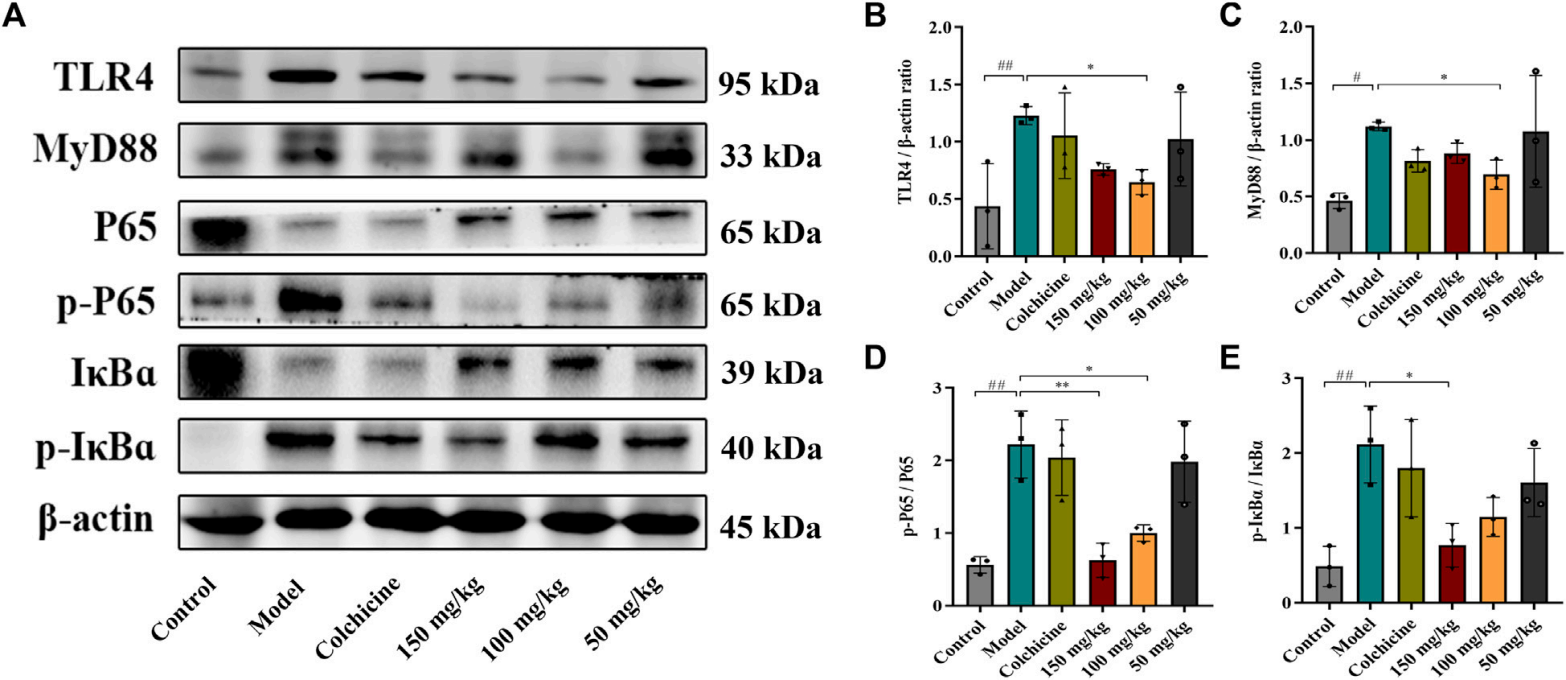
Western blotting photographs illustrating protein levels of TLR4/MyD88/NF-κB signaling pathway. **(A–E)** The expressions and quantitative analysis of TLR4, MyD88, p-P65/P65 and p-IκBɑ/IκBɑ proteins (*n* = 3). Values were presented as the means ± SD. ^*#*^ *p* < 0.05, ^*##*^ *p* < 0.01 vs. control group; ^*^ *p* < 0.05, ^**^ *p* < 0.01 vs. model group.

**FIGURE 6:**
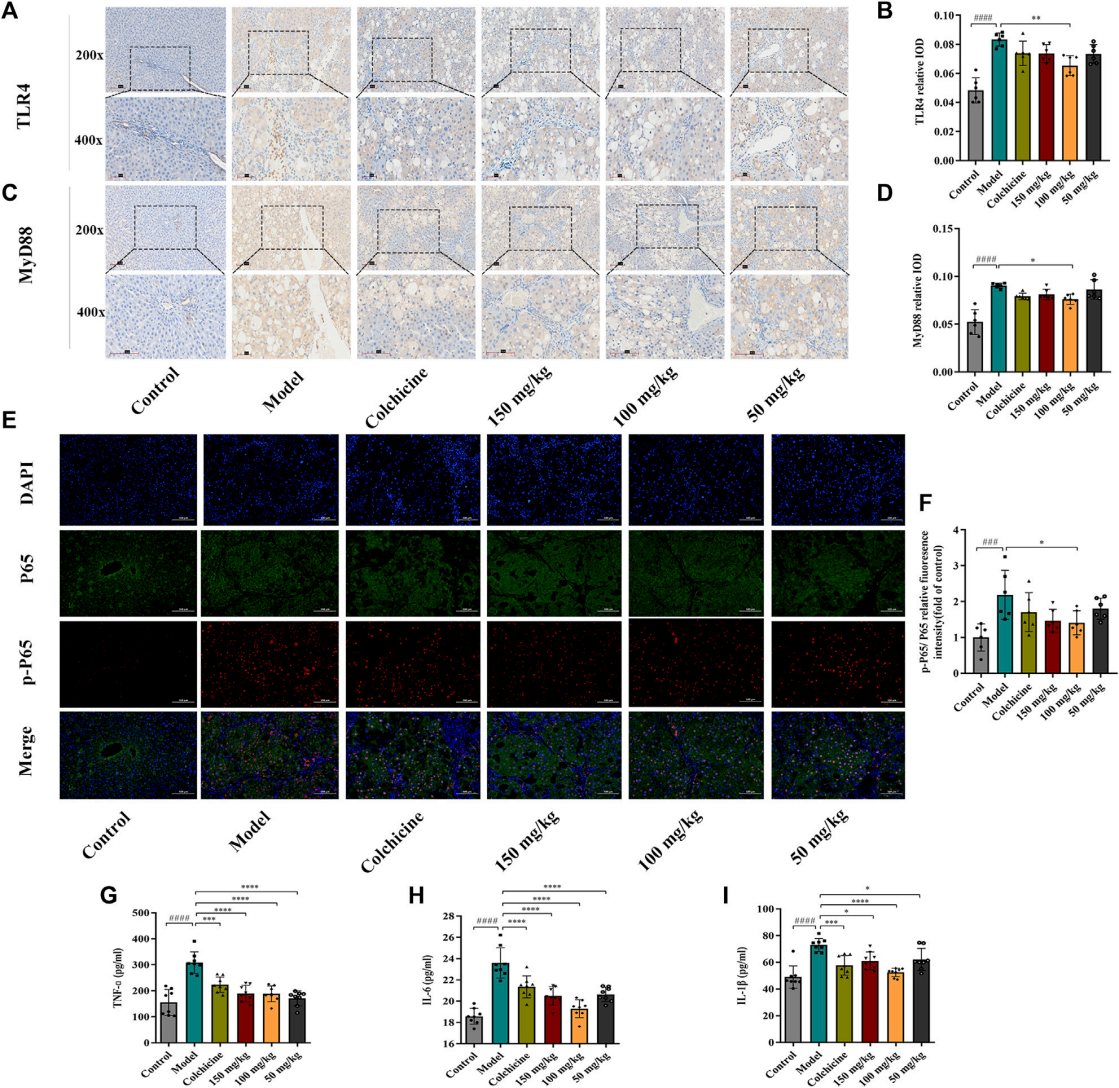
The expression of TLR4, MyD88, p-P65/P65 in liver tissue and the level of inflammatory cytokines in serum. **(A–D)** The expressions of TLR4 and MyD88 by immunohistochemical analysis (*n* = 6). **(E–F)** The expressions of p-P65/P65 by immunofluorescence staining analysis (*n* = 6). **(G–I)** The content of TNF-ɑ, IL-6 and IL-1β in serum (*n* = 8). Values were presented as the means ± SD. ^###^ *p* < 0.001, ^####^ *p* < 0.0001 vs. control group; ^*^ *p* < 0.05, ^**^ *p* < 0.01, ^***^ *p* < 0.001 and ^****^ *p* < 0.0001 vs. model group.

**FIGURE 7:**
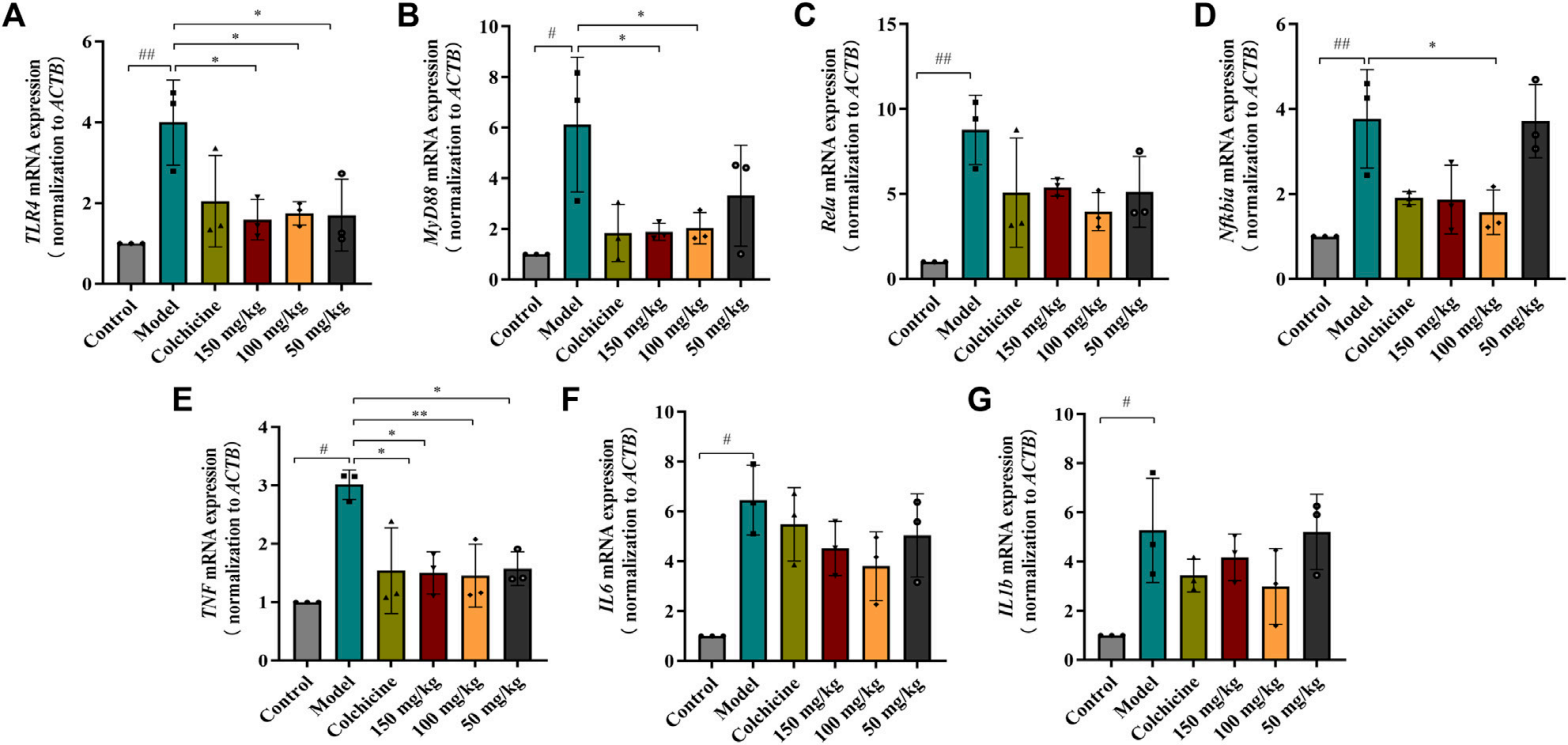
RT-qPCR analysis the mRNA expression of TLR4/MyD88/NF-κB signaling pathway. **(A–D)** The expressions of *TLR4*, *MyD88*, *Rela* and *Nfkbia* mRNA (*n* = 3). **(E–G)** The expressions of *TNF*, *IL6* and *IL1b* mRNA in tissue (*n* = 3). Values are presented as the means ± SD. ^#^ *p* < 0.05, ^##^ *p* < 0.01 vs. control group; ^*^ *p* < 0.05, ^**^ *p* < 0.01 vs. model group.

Myofibroblast activation and fibrogenesis in the liver are both driven by TLR4. Furthermore, it has been demonstrated that the TLR4-dependent regulation of TGF-β signaling acts as a link between pro-inflammatory and profibrogenic signals (Seki et al., 2007). Consequently, we further investigated whether the anti-fibrotic effects of TFPCP involved the regulation of inflammation. Western blot analysis showed that the expression of TLR4 and MyD88 was markedly downregulated following TFPCP treatment, while the p-P65/P65 and p-IκBα/IκBα ratios were notably decreased relative to the Model group (Figures 5A–E). Simultaneously, immunohistochemical and qPCR assays further demonstrated that TFPCP inhibited the expression of TLR4 and MyD88 (Figures 6A–D; Figures 7A, B). The mRNA levels of *Rela* and *Nfkbia* were also markedly increased in the Model group (Figures 7C, D) compared with those in the Control group, whereas the opposite was seen with TFPCP treatment. In addition, we performed double immunofluorescence staining for P65 and p-P65 (Figure 6E) and found that the p-P65/P65 ratio (Figure 6F) was significantly decreased in the TFPCP treatment group relative to that in the Model group. Meanwhile, to explore the role of TFPCP in regulating fibrogenic responses in the liver, pro-inflammatory cytokine levels were evaluated by ELISA and RT-qPCR. As shown in Figures 6G–I; Figures 7E–G, CCl_4_ treatment led to a noticeable increase in TNF-α, IL-6, and IL-1β contents in both serum and liver tissue compared with saline-only treatment (Control group). In contrast, the TNF-α, IL-6, and IL-1β level in serum were significantly lower in the TFPCP group than in the Model group. While, in addition to *TNF* mRNA, the expression of *IL6* and *IL1b* mRNA level was also lower in the TFPCP treatment groups than in the Model group, but the difference was not significant (Figures 7F, G). Taken together, these results suggested that TFPCP can limit the inflammatory response induced by CCl_4_, thereby alleviating TLR4/MyD88/NF-κB signaling pathway-mediated hepatic fibrogenesis.

### 3.6 TFPCP regulated hepatic fibrosis-related metabolites

**FIGURE 8:**
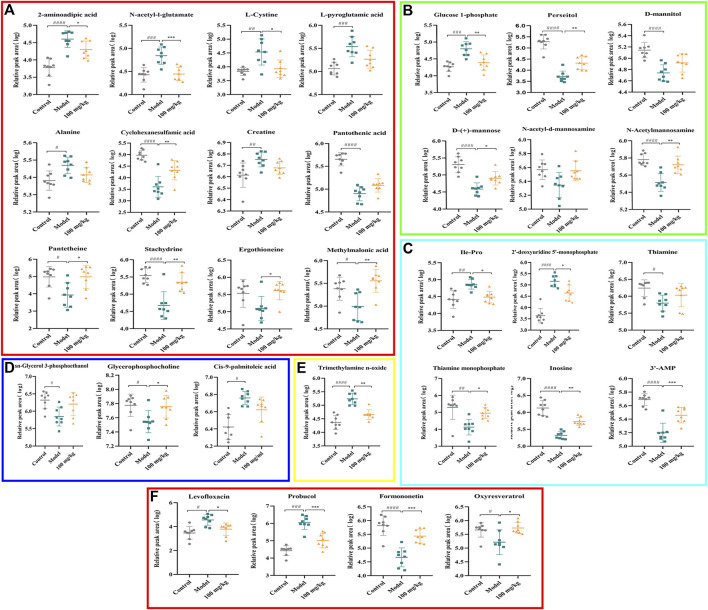
Effects of TFPCP on hepatic fibrosis related differential metabolites. **(A)** Effect of TFPCP-M (100 mg/mL) on amino acids metabolism (*n* = 8); **(B)** Effect of TFPCP-M (100 mg/mL) on carbohydrates metabolism (*n* = 8); **(C)** Effect of TFPCP-M (100 mg/mL) on nucleosides metabolism (*n* = 8); **(D)** Effect of TFPCP-M (100 mg/mL) on lipids metabolism (*n* = 8); **(E)** Effect of TFPCP-M (100 mg/mL) on trimethylamine n-oxide (TMAO) metabolism (*n* = 8); **(F)** Other metabolism (*n* = 8).

**FIGURE 9:**
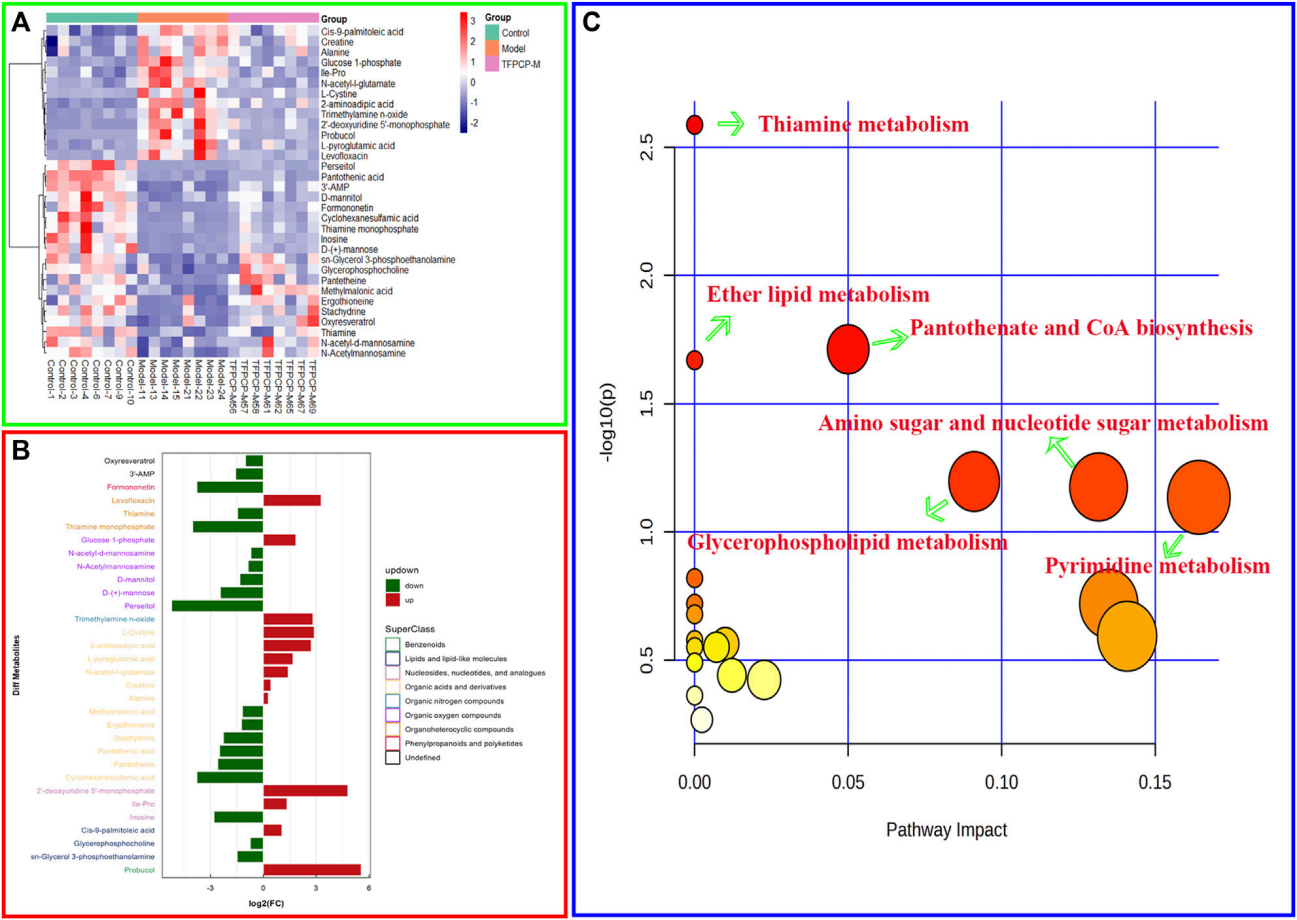
Effects of TFPCP on hepatic fibrosis related metabolic pathways. **(A)** Heatmap for the effect of TFPCP-M (100 mg/mL) supplementation on tissue metabolites in CCl_4_-induced hepatic fibrosis rats (*n* = 8). **(B)** Multiple analysis of significant difference metabolite expression (*n* = 8). **(C)** The enrichment pathways result in the production of 32 metabolites.

The PCA and OPLS-DA score diagrams for the positive and negative ion types are shown in Supplementary Figure S2. Significant clustering could be detected among the different groups. Metabolites meeting the VIP >1 and *p* < 0.05 criteria were considered to be differentially abundant. A total of 32 distinct metabolites were identified when the metabolomic analysis was performed in both positive and negative ion modes (Table 3). Nevertheless, after TFPCP administration, the levels of 20 of the 32 distinct metabolites were significant restored (Figures 8A–F; Figure 9A). Meanwhile, the pathways that overlapped between the Control group and the Model group, and between the Model group and the TFPCP group, were regarded as relevant pathways. The differentially abundant metabolites were primarily involved in lipid, energy, nucleoside, and amino acid metabolism (Figure 9B), and could be categorized into the following six major metabolic pathways (Figure 9C): Thiamine metabolism, Ether lipid metabolism, Pantothenate and CoA biosynthesis, Amino sugar and nucleotide sugar metabolism, Glycerophospholipid metabolism, and Pyrimidine metabolism.

**TABLE 3:** Results of the discovery of putative biomarkers in rat tissue.

No.	Retention time (min)	m/z	Metabolite	Ionization Mode	*p*-value	VIP	HMDB ID	Trend Model/Control	Trend Model/Md
1	47.616	253.2135	Cis-9-palmitoleic acid	[M-H]-	2.17E-05	10.5792	HMDB0003229	↑	↓
2	414.004	160.0583	2-aminoadipic acid	[M-H]-	8.01E-05	1.08361	HMDB0000510	↑	↓
3	122.5085	227.0636	Ile-Pro	[M-H]-	0.000517	1.236435	HMDB0000012	↑	↓
4	92.049	307.0197	2′-deoxyuridine 5′-monophosphate	[M-H]-	0.00053	2.185498	HMDB0001409	↑	↓
5	406.521	241.0075	Glucose 1-phosphate	[M-H-H2O]-	0.000746	1.218041	HMDB0001586	↑	↓
6	396.295	188.0527	N-acetyl-l-glutamate	[M-H]-	0.000926	1.146512	HMDB0001138	↑	↓
7	305.832	128.0372	L-pyroglutamic acid	[M-H]-	0.002144	2.785282	HMDB0000267	↑	↓
8	57.0015	316.1298	Levofloxacin	[M-H-CO2]-	0.004883	1.142444	HMDB0001929	↑	↓
9	469.136	239.0124	L-Cystine	(M-H)-	0.01528	1.030341	HMDB0000192	↑	↓
10	397.191	76.07501	Trimethylamine n-oxide	[M + H]+	0.00022	1.344752	HMDB0000925	↑	↓
11	409.603	132.0769	Creatine	[M + H]+	0.003934	4.945975	HMDB0000064	↑	↓
12	409.411	90.05446	Alanine	[M + H]+	0.004155	1.061756	HMDB0000161	↑	↓
13	282.197	437.2081	Pantothenic acid	[2M-H]-	1.19E-06	3.079242	HMDB0000210	↓	↑
14	88.908	357.0956	Cyclohexanesulfamic acid	[2M-H]-	0.000132	1.52676	HMDB0031340	↓	↑
15	337.734	211.0781	Perseitol	[M-H]-	0.000134	2.244984	HMDB0033750	↓	↑
16	218.851	267.0694	Inosine	[M-H]-	0.000255	5.442514	HMDB0000195	↓	↑
17	301.6545	181.0671	D-mannitol	[M-H]-	0.000443	1.319364	HMDB0000765	↓	↑
18	307.886	359.1142	D-(+)-mannose	[2M-H]-	0.000554	2.015165	HMDB0062473	↓	↑
19	90.059	343.0805	Thiamine monophosphate	[M-H]-	0.001985	2.602537	HMDB0002666	↓	↑
20	394.124	214.0522	sn-Glycerol 3-phosphoethanolamine	[M-H]-	0.002616	4.907646	HMDB0000114	↓	↑
21	74.548	277.119	Pantetheine	(M-H)-	0.004156	1.455189	HMDB0003426	↓	↑
22	397.737	117.0217	Methylmalonic acid	[M-H]-	0.009969	1.349379	HMDB0000202	↓	↑
23	325.296	204.0852	N-Acetylmannosamine	(M + H-H2O)+	3.57E-05	1.068466	HMDB0001129	↓	↑
24	406.4905	265.1104	Thiamine	[M]+	0.000943	2.58757	HMDB0000235	↓	↑
25	341.853	144.1004	Stachydrine	[M + H]+	0.001233	1.418089	HMDB0004827	↓	↑
26	447.009	258.1107	Glycerophosphocholine	[M + H]+	0.007729	6.920794	HMDB0000086	↓	↑
27	390.619	230.0945	Ergothioneine	[M + H]+	0.044483	1.138287	HMDB0003045	↓	↑

↑: upregulated. ↓: downregulated.

↑: compared to the control group, the compound’s level was elevated in the model group.

↓: compared to the model group, the compound’s level was reduced in intervention group.

## 4 Discussion

Fibrosis, characterized by the net buildup of ECM and scarring, has been recognized for decades in patients with chronic liver illnesses. Throughout the majority of medical history, fibrosis was thought to be intractable (Lee et al., 2015). Despite extensive research into hepatic fibrosis (Kisseleva and Brenner, 2021), no anti-fibrotic medicines has yet been authorized for this condition. Natural products have gained substantial interest as novel anti-fibrotic medications given their rich diversity of chemical structure, biological activity, and drug-like characteristics. PCP has long been widely employed as an effective treatment for a variety of liver conditions in China. More recently, PCP has been shown to possess hepatoprotective properties in both *in vivo* and *in vitro* settings, effects that may be mediated by TFPCP. In this study, using pharmacodynamic analysis, we demonstrated that this herbal extract exerted preventive and alleviatory effects on hepatic fibrogenesis induced by CCl_4_. Mechanistically, we found that TFPCP could mitigate inflammation, thus alleviating TLR4/MyD88/NF-κB signaling pathway-mediated hepatic fibrogenesis. The metabolomic analysis resulted in the identification of 32 distinct metabolites corresponding to 22 metabolic pathways. The levels of 20 of these metabolites were greatly restored with TFPCP treatment. These 32 metabolites were mainly involved in lipid, energy, nucleoside, and amino acid metabolism, and were likely to be associated with the occurrence of hepatic fibrosis.

The UHPLC–Q-Orbitrap HRMS analysis of TFPCP revealed the presence of 14 flavonoids, including quercetin, taxifolin, pinocembrin, and luteoloside, among others. Many of these flavonoids exhibit a wide range of pharmacological properties, such as antioxidant, anti-inflammatory, and antimicrobial effects (Chirumbolo and Bjorklund, 2018; Shang et al., 2021). In this study, we provided further evidence that TFPCP can ameliorate liver injury and fibrosis. In the clinic, ultrasound is widely used for diagnosing the staging of liver fibrosis and assessing therapeutical effects. Here, ultrasound analysis showed that CCl_4_ treatment led to marked increases in gray values and the DDPV and a decrease in VPV values. Additionally, the liver tissue of animals in the Model group exhibited an irregular arrangement of hepatocytes, thin fibrous septa, and round vacuoles of varying sizes in the cytoplasm, as evidenced by the H&E staining results. The increases in the levels of ALT, AST, TBIL, ALB, ALP, and TP in serum provided more evidence that CCl_4_ can cause liver impairment and dysfunction. However, the oral administration of TFPCP resulted in a substantial improvement in ultrasound parameters and pathological changes, as well as the downregulation of serum levels of biochemical indexes, indicating that TFPCP can ameliorate CCl_4_-induced liver damage. During hepatic fibrogenesis, activated HSCs are the primary producers of excess ECM, including collagen (Li et al., 2018). Meanwhile, TGF-β_1_ is regarded as the most potent profibrogenic cytokine and is also a key mediator of HSC activation *in vitro* and *in vivo* (Seki et al., 2007). Its effects include the stimulation of myofibroblasts, the induction of the synthesis of ECM components, and the suppression of collagen degradation. In this study, we found that TFPCP reduced collagen deposition and HSC activation compared with the Model group, as evidenced by the results of Masson staining, tissue TGF-β_1_ immunohistochemical analysis, and assessment of serum TGF-β_1_ contents (Figure 4). Combined with the decrease in the levels of PC III, COL4, LN, and HA in serum, these findings support that TFPCP treatment can alleviate CCl_4_-induced liver fibrosis.

In this study, the therapeutic effect of TFPCP on liver fibrosis did not show a dose-dependent relationship. The effect of the 150 mg/kg was not better than that of the 100 mg/kg. This phenomenon is quite common in TCM pharmacology due to the multiple ingredients and multiple targets as stated in the textbook (Peng, 2016). The drug TFPCP is a cluster of flavonoids from PCP, which includes more than 14 different compounds as detected by the UHPLC–Q-Orbitrap HRMS analysis in the study. Therefore, the absence of dose dependence here may be also caused by multiple components of TFPCP with different activities. In particular, we suspect it is because of the ingredients that can modulated the activity of drug metabolizing enzymes or transporters. P-glycoprotein is one of the main efflux transporters proteins in human body, which excretes exogenous substances such as poisons and drugs out of the cells and functions as a barrier for the body (Conseil et al., 1998). Cytochrome P450 (CYP) enzymes are involved in the oxidative biotransformation of most drug (Meunier et al., 2004). CYP3A4 takes part in the biotransformation of more than 50% of the orally administered medications, and some other CYP isoforms (Kaci et al., 2023). Organic anion transporting polypeptides (OATPs) are solute carrier-type membrane transporters, which are commonly involved in the tissue uptake of nutrients, drugs, and toxins (Mandery et al., 2012). The induce of CYP-catalyzed elimination and/or inhibition of OATP-mediated transport of drugs commonly lead to the development of pharmacokinetic interactions. For instance, It is found flavones (such as quercetin) were indeed shown to bind to purified P-glycoprotein and to efficiently inhibit its activity more strongly than flavanones (naringenin) (Conseil et al., 1998), and following it may reduce the excretion of other chemical components (naringenin). However, study reveals that the high intake of luteolin and naringenin can lead to the *in vivo* inhibition of hepatic and intestinal OATP2B1 and/or OATP1B1transporters mediated absorption of certain drugs (Kaci et al., 2023). In addition, quercetin can increase the expression of CYP3A4 in human hepatocytes, resulting in an induction effect (Raucy, 2003), thereby reducing drug bioavailability by increasing drug efflux and drug metabolism in the intestine and liver. Therefore, we speculate that in the high dose (150 mg/kg), there are more flavones in the drug induces strong influence on expression of CYP3A4 in liver, as well as binding to P-glycoprotein and OATP-mediated transport, which would finally reducing drug bioavailability for the active ingredients that protecting liver.

**FIGURE 10:**
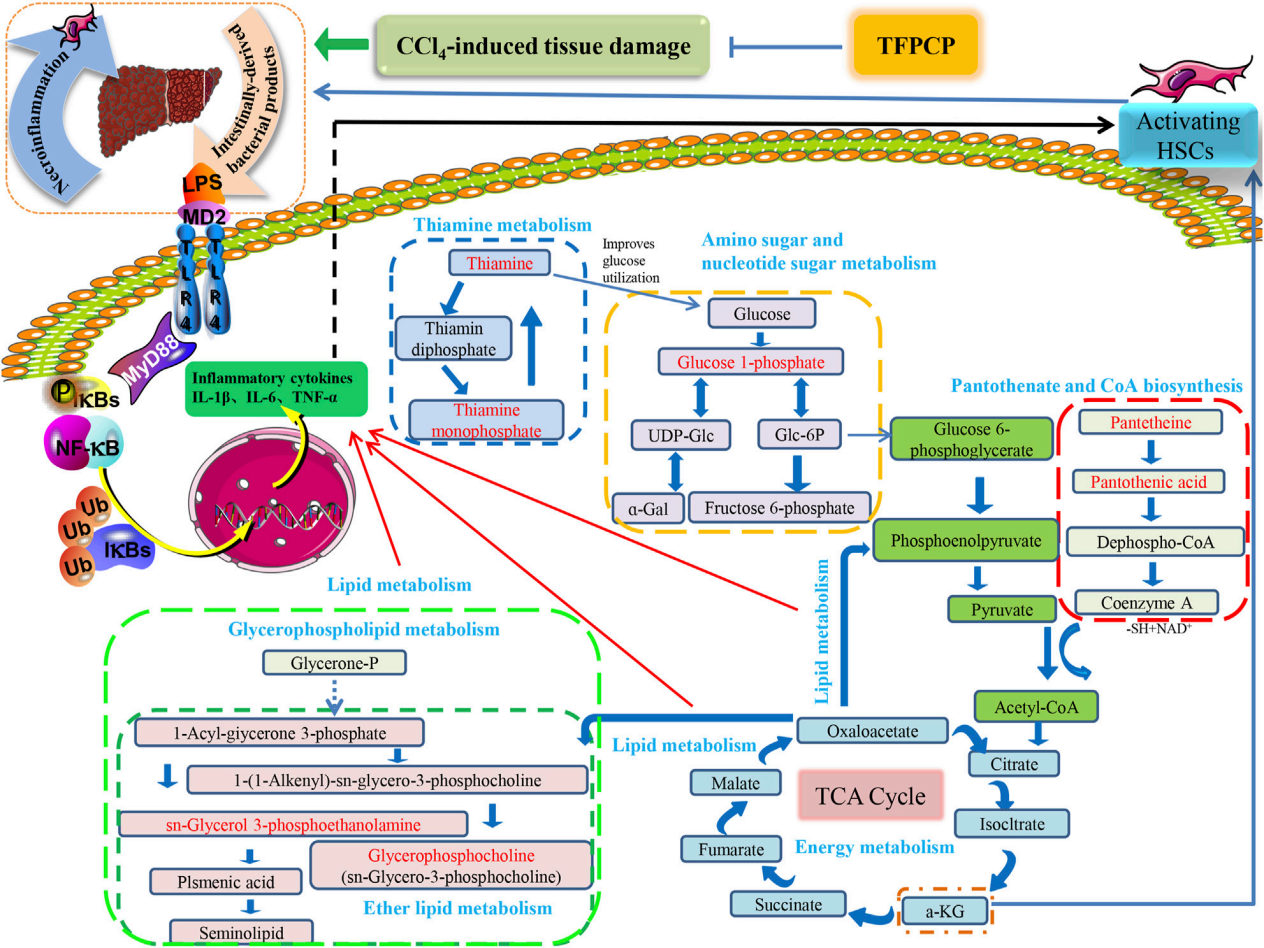
The relationship between differential metabolic pathways and the correlation analysis with the pharmacological effects of TFPCP on CCl_4_-induced hepatic fibrosis rats, and the red portion represents the various metabolites studied in our investigation.

Tissue damage caused by toxins such as CCl_4_ usually results in cell death. Necrotic cells and damaged tissues release inflammatory stimuli, many of which are categorized as danger-associated molecular patterns. The binding of these factors to their corresponding “pattern recognition receptors”, such as TLRs, results in the release of pro-inflammatory factors such as TNF-α, IL-6, and IL-1β. This can lead to the activation of NF-κB, resulting in the continuous amplification of the initial inflammatory signals and the so-called inflammatory cascade effect. Meanwhile, the LPS/TLR4 signaling pathway is primarily responsible for mediating the inflammatory response and pro-fibrogenic activity in numerous liver diseases, and studies have established that this route contributes to liver damage and fibrosis (Nobili et al., 2015; Mack, 2018; Unsal et al., 2021). Consequently, we next focused on investigating one of the most common signal transduction pathways of the TLR4/NF-κB signaling system, that is, the MyD88-dependent pathway. We found that the expression of TLR4 and MyD88 and the p-P65/P65 and p-IκBα/IκBα ratios were noticeably downregulated in liver tissue after TFPCP intervention. Meanwhile, TGF-β can synergize with IL-6, TNF-α, or IL-1β to accelerate the development of hepatic fibrosis (Kisseleva and Brenner, 2021). In our study, the serum and tissue contents of TNF-α, IL-6, and IL-1β were lower in the TFPCP group than in the Model group as determined by ELISA and RT-qPCR (Figures 6G–I; Figures 7E–G). These results are compatible with the finding that treatment with TFPCP reduced the levels of the pro-inflammatory mediator TGF-β_1_. Here, we provided evidence that TFPCP can mitigate the inflammatory response by regulating the TLR4/MyD88-mediated NF-κB signaling pathway, thereby ultimately inhibiting hepatic fibrogenesis (the putative signaling pathway is depicted in Figure 10).

Hepatic fibrosis is a metabolic disorder characterized by the presence of a multitude of aberrantly expressed endogenous metabolites. Thus, in this study, we used UHPLC-Triple TOF-MS/MS-based metabolomics to investigate the hepatic fibrosis-related liver tissue metabolite profile. The liver is an essential organ for lipid metabolism, and lipid metabolism is frequently impaired in conditions that affect the liver (Weber et al., 2003). Specifically, CCl_4_ is transformed into CCl_3_• by the cytochrome P450-dependent mixed-function oxidase system. This radical can react with oxygen to produce the trichloromethylperoxy radical (CCl_3_OO•), which then initiates a lipid peroxidation cascade, resulting in the destruction of polyunsaturated fatty acids, particularly those associated with phospholipids (Weber et al., 2003; Usami et al., 2013). Meanwhile, organic acids are known to exert a strong effect on lipid metabolism (Usami et al., 2013), and fatty acid production has been linked to energy metabolism, anti-inflammatory qualities, and antioxidant capabilities (Tang et al., 2022). Thus, metabolic disorders involving lipids and lipid-like molecules are extremely influential in the development of liver fibrosis.

Glycerophospholipids (GPs), as storage deposits for lipid mediators, function as integral membrane proteins, transporters, receptors and ion channels (Lieber et al., 1994). Therefore, dysfunction of the glycerophospholipid metabolism could negatively affect the energy metabolism in the liver (Mesens et al., 2012). Published data also reported CCl_4_-treated rats can be subjected to perturbations of lipid metabolism to induce liver fibrosis (Chang et al., 2017). In animal cells, glycerophosphocholine (GroPCho) is formed via the deacyllation of the phospholipids Phosphatidylcholine (PC), which play a key role in the architecture of eukaryote membranes. Sn-Glycerol 3-phosphoethanolamin (GroPEtn) is a breakdown product of phosphatidylethanolamine (PE), and present in higher levels in normal liver relative to other organs (Tallan et al., 1954), which could stimulate the growth of hepatocytes and dropped significantly during liver regeneration (B Gowda et al., 2020). The decreased of PC to PE ratio impaired the cell membrane component and induced the permeability of the hepatocytes, which accelerated liver injury (Calzada et al., 2016). Also, altered PC/PE ratio has been shown to influence the dynamics and regulation of lipid droplets contributing towards steatosis (Mainali et al., 2021). Consistently, our results showed that the levels of GroPCho and GroPEtn decreased in the fibrosis rat model, and massive hepatocyte steatosis were found in the results of H&E pathological staining. On the contrary, TFPCP treatment increased the level of GroPCho and GroPEtn in liver tissue, alleviated liver damage and improved the pathology change, indicating that GPs metabolism was disturbanced in the process of liver fibrosis.

Thiamine is one of the carbohydrates metabolizing co-enzymes stored mainly in the liver (Hassan et al., 1991). Published research have shown that the cirrhotic liver may directly or indirectly affect phosphorylation, resulting in decreased levels of diphosphothiamine and, thus, hepatic thiamine storage (Hassan et al., 1991). Therefore, thiamine deficiency is likely to produce inadequate glucose utilization (Hassan et al., 1991). Meanwhile, research have shown animal models of thiamine deficiency have revealed increases in the levels of pro-inflammatory markers such as TNF-α (Karuppagounder et al., 2007). Similarly, our results showed that TFPCP could ameliorate the inflammatory response (TNF-α, Il-6 and IL-1β) in a CCl_4_-induced liver fibrosis rat model. Furthermore, the level of Thiamine, and its phosphate derivatives, thiamine monophosphate were decreased in the Model group, while TFPCP treatment increased these two biomarker levels.

In the Pantothenate and CoA biosynthesis pathway, the biomarker of pantothenic acid (vitamin B5), is the synthetic materials for coenzyme A. and pantetheine is the cysteamine amide analog of pantothenic acid which plays a central role in energy metabolism (Zhao et al., 2021). On the one hand, inflammation is energetically expensive (Zhang et al., 2022). Similarly, the level of pro-inflammatory markers TNF-α, IL-6 and IL-1β were correspondingly decrease in the TFPCP treatment groups than Model group. On the other hand, published research shown that pantothenic acid is a profibrotic agent that may increase and accelerate the wound-healing processes by recruiting migrating fibroblasts to the affected areas and promoting the proliferation and activation of fibroblasts, and collagen synthesis (Mariani and Roncucci, 2017). However, the changes in these two biomarkers in our experiments are not consistent with research reports, and we speculate that this may be the result of prolonged and not equilibrated succession of proliferation and death can lead to erosion of the liver cells, and thus to lack of function of the physical barrier.

Meanwhile, it is well established that metabolic processes involving galactose, nucleotide sugars, and amino sugars are also linked to chronic inflammation (Mir et al., 2022). Research reported that almost all glucosyl transfer reactions rely on glucose-1-phosphate (Glc-1-P) that either immediately acts as glucosyl donor or as substrate for the synthesis of the more widely used Glc dinucleotides, ADPglucose or UDPglucose (Fettke et al., 2011). Interestingly, in our research, 10 of the 32 metabolites that participate in amino and nucleotide sugar metabolism were restored after treatment with TFPCP (Figure 9A). This is in line with our finding that serum levels of pro-inflammatory factors were increased in hepatic fibrosis model rats. The results of the metabolomic analysis further confirmed that TFPCP inhibited the inflammatory response by regulating energy, lipid, nucleoside, and amino acid metabolism, thereby protecting against tissue damage and exerting anti-hepatic fibrosis effects (Figure 10).

In summary, employing a combination of molecular biology and metabolomic techniques, we elucidated the mechanism underlying the anti-hepatic fibrosis effects of TFPCP. We found that they are associated with the inactivation of the TLR4/MyD88/NF-κB signaling pathway and the regulation of lipid, energy, nucleoside, and amino acid metabolism. These findings suggest that TFPCP has potential as a natural therapy for hepatic fibrosis.

## Data Availability

The original contributions presented in the study are included in the article/Supplementary Material, further inquiries can be directed to the corresponding authors.

## Supplementary material

SUPPLEMENTARY FIGURE S1 Total ion chromatogram of TFPCP extract in positive-ion mode **(A)** and negative-ion mode **(B).**

SUPPLEMENTARY FIGURE S2 Multivariate analysis of liver tissue metabolomics. **(A, B)** PCA score plot of liver tissue samples collected from different treatment groups of rats in positive and negative ion mode. **(C–F)** The difference distribution of tissue metabolites between control and model group by OPLS-DA and permutation tests score analysis in positive and negative ion mode. **(G–J)** The difference distribution of tissue metabolites between model and TFPCP-M (100 mg/mL) group by OPLS-DA and permutation tests score analysis in positive and negative ion mode.

## Abbreviations

ALB, Albumin; ALP, Alkaline phosphatase; ALT, Alanine aminotransferase; AST, Aspartate aminotransferase; BSA, Bovine Serum Albumin; CCl4, carbon tetrachloride; COL4, Collagen Type Ⅳ; CYP, Cytochrome P450; ECM, extracellular matrix; GroPCho, glycerophosphocholine; GroPEtn, Sn-Glycerol 3-phosphoethanolamin; GPs, Glycerophospholipids; HA, Hyaluronidase; H&E, hematoxylin & eosin; HPLC high-performance liquid chromatography; HSCs, hepatic stellate cells; IL-1β, interleukin 1 Beta; LN, Laminin; MyD88, myeloid differentiation factor 88; OATPs, Organic anion transporting polypeptides; PCP, Penthorum chinense Pursh; PC III, Procollagen III; TBIL, Total bilirubin; TFPCP, Total flavonoids of Penthorum chinense Pursh; TNF-α, Tumor necrosis factor-alpha; TP, Total protein.
