# Supplementary figures and images for: Total flavonoids extracted from Penthorum chinense Pursh mitigates CCl4-induced hepatic fibrosis in rats via inactivation of TLR4-MyD88-mediated NF-κB pathways and regulation of liver metabolism
Source: Front Pharmacol. 2023 Nov 23;14:1253013. doi: 10.3389/fphar.2023.1253013 (PMC10701287; doi:10.3389/fphar.2023.1253013)

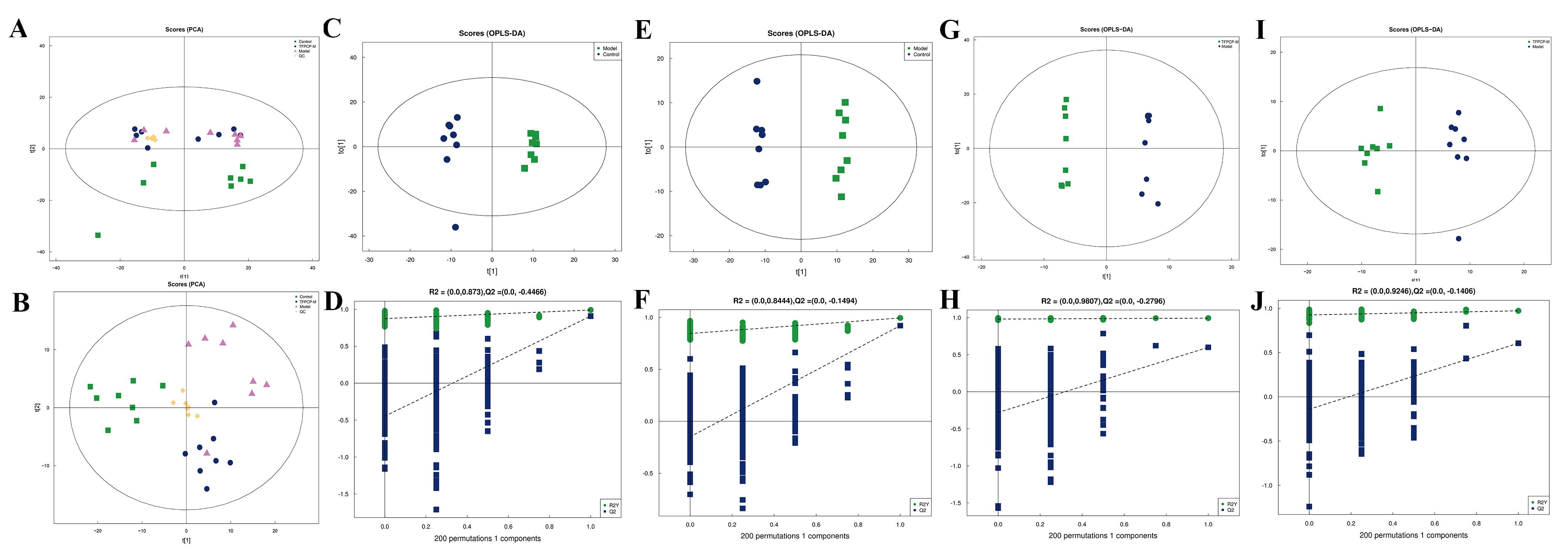

Supplement: Supplementary file 1 [file Image2.TIF]

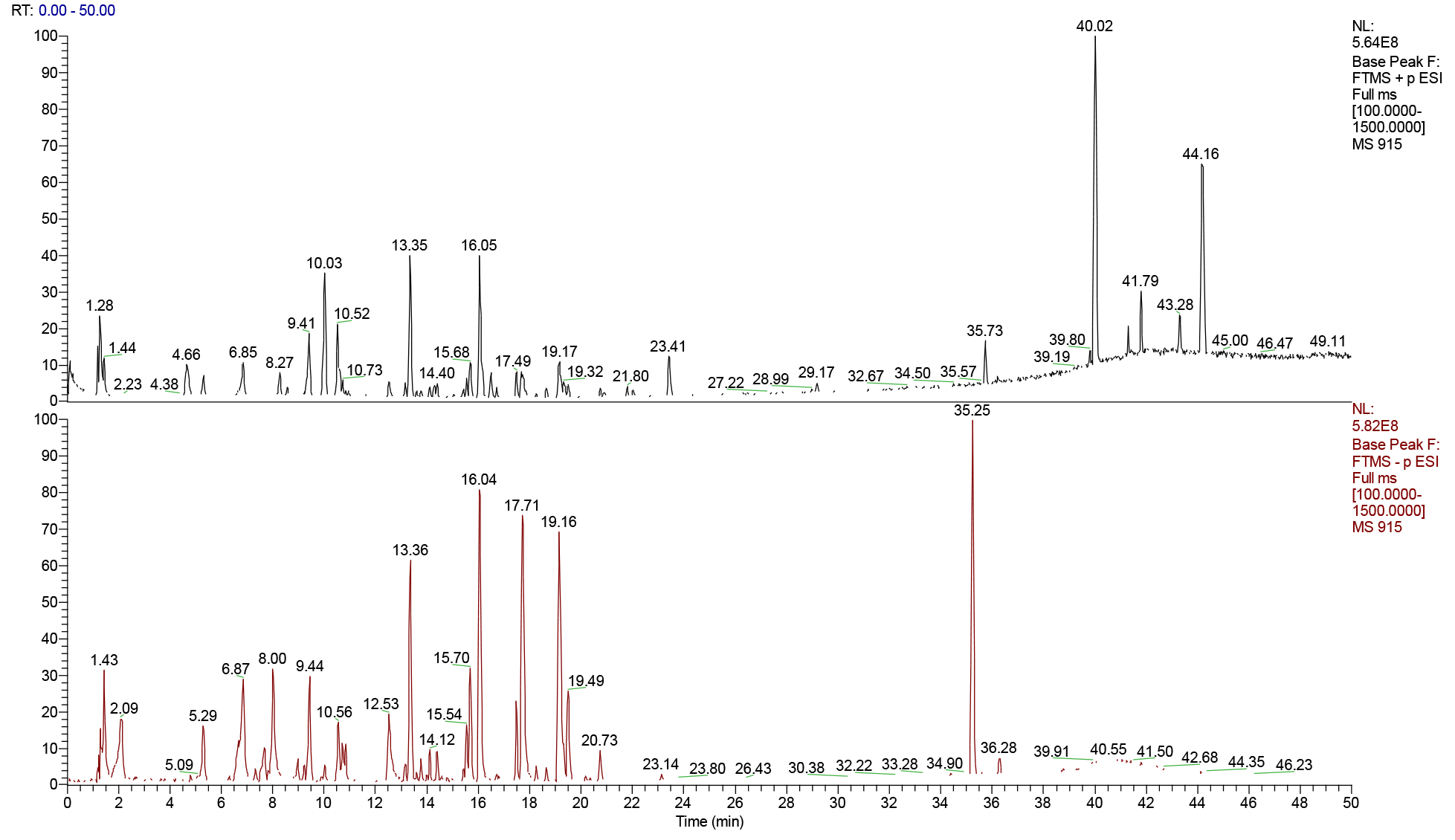

Supplement: Supplementary file 2 [file Image1.TIF]
